# Wheat Yellow Mosaic Virus P1 Inhibits ROS Accumulation to Facilitate Viral Infection

**DOI:** 10.3390/ijms26041455

**Published:** 2025-02-10

**Authors:** Yingjie Zhao, Jiaqian Yang, Ying Liu, Xiaodi Hu, Xia Wang, Jian Yang, Jiaqian Liu

**Affiliations:** 1 State Key Laboratory for Managing Biotic and Chemical Threats to the Quality and Safety of Agro-Products, Key Laboratory of Biotechnology in Plant Protection of MARA, Key Laboratory of Green Plant Protection of Zhejiang Province, Institute of Plant Virology, Ningbo University, Ningbo 315211, China; 2 Institute of Mass Spectrometry, Zhejiang Engineering Research Center of Advanced Mass Spectrometry and Clinical Application, School of Material Science and Chemical Engineering, Ningbo University, Ningbo 315211, China

**Keywords:** BSMV-VIGS, RNA-seq and LC-MS/MS, ROS scavenging, WYMV-P1

## Abstract

Reactive oxygen species (ROS), as signaling molecules, play a crucial role in the plant immune response. However, the mechanism(s) by which viruses affect ROS metabolism remain largely unexplored. Here, we found that wheat yellow mosaic virus (WYMV)-encoded P1 is a pathogenic protein. Transcriptomic and proteomic integrative analyses were performed on WYMV-infected overexpressing-P1 wheat and wild-type plants. A total of 9245 differentially expressed genes (DEGs) and 1383 differentially expressed proteins (DEPs) were identified in the transcriptome and proteome, respectively. At their intersection, 373 DEGs/Ps were identified. Enrichment analysis revealed that the expression of genes related to the ROS metabolism pathway in overexpressed P1 transgenic wheat (OE-P1) plants significantly increased during WYMV infection. We screened peroxidase (TaPOD) and thioredoxin reductase (TaTrxR) as they showed the most significant differences in expression. The silencing of *TaPOD* and *TaTrxR* revealed that they positively regulate WYMV infection by reducing ROS accumulation. Furthermore, hydrogen peroxide treatment induced WYMV resistance in wild-type wheat plants and OE-P1 transgenic plants. This study provides a theoretical basis for the role of P1 in plant viral infection.

## 1. Introduction

Viral infections typically disrupt plant defenses, such as reactive oxygen species (ROS)-, salicylic acid (SA)-, and jasmonic acid (JA)-mediated defenses [1,2,3]. Disruption of these pathways indicates that the plant’s immune response is affected. It has long been known that viral infections cause significant changes in gene expression in these pathways [4]. The role of ROS in plant defense against viral infections has been studied but also remains largely unknown [5,6,7].

ROS are essential signaling molecules in the process of plant development and the stress response [8,9,10,11]. In addition, ROS play important roles in plant antiviral defense [12,13]. However, high concentrations of ROS can disrupt normal cell function, leading to oxidative damage. In response to excess ROS, plants are equipped with a complex antioxidant defense system consisting of enzymatic and nonenzymatic components that either clear ROS or inhibit their harmful effects [14]. For example, increased expression of superoxide dismutase (SOD), catalase (CAT), and peroxidase (POD) reduces the effects of ROS in plants. Arabidopsis and rice contain three NADPH-dependent thioredoxin reductases (NTRs) that transfer reducing capacity to the thioredoxin/peroxidase (Trx/Prx) system to scavenge ROS [15].

RNA sequencing (RNA-Seq) is the most commonly used and powerful tool for analyzing differentially expressed genes [16]. However, the proteome can reflect the actual expression of the proteins corresponding to these genes. To elucidate the effects of changes from the gene level to the protein level on plant immunity, integrated transcriptome–proteome interaction analysis is essential. For example, transcriptomic and proteomic analyses revealed gene expression patterns unique to the grapevine fanleaf virus symptom determinant residue 802 of the protein 1EPol [17]. Integrated proteomic and transcriptomic analyses were employed to identify pathogen-associated protein genes by comparing the differential expressed proteins (DEPs) and genes (DEGs) in maize after inoculation with the fungus *F. verticillioides* [18].

Wheat (*Triticum aestivum*) is one of the world’s most important staple food crops [19]. It usually receives various biotic and abiotic stresses during growth stages. In China, wheat yellow mosaic virus (WYMV) diseases can lead to yield reductions of up to 70% in severe cases [20]. Additionally, 15 °C is the most favorable temperature for WYMV infection while 8 °C is conducive to the pathogenesis of WYMV [21]. WYMV belongs to the genus *Bymovirus* in the family *Potyviridae* [20]. WYMV’s genome contains two single-stranded RNAs. Among them, RNA1 is 7.6 kb in length and encodes a polyprotein of 270 kDa. After protease treatment, this polyprotein produces nine mature proteins to perform the different functions. RNA2 is 3.5 kb in length and encodes a polyprotein of 100 kDa. After enzymatic digestion, this polymeric protein produces two mature proteins, P1 and P2. Both of these proteins function as viral RNA silencing suppressors [22,23]. During WYMV infection, P1 plays the role of a viral suppressor of RNA silencing (VSR) by interfering with calmodulin-related antiviral RNAi defense mechanisms. The structural analysis of the P1 revealed a striking similarity to the carboxyl terminus of the HC-Pro protein [24]. In addition, P1/HC-Pro can regulate the synthesis of abscisic acid in the host. In the P1 proteins of other viruses, there are also diverse functions. For example, sugarcane stripe mosaic virus P1 weakened the antiviral immunity of plants and increased the infection of the potato virus X in *N. benthamiana* [25]. The P1 of rice yellow mottle virus can replicate independently and move locally in both host and non-host plants [26]. These findings suggest that P1 may be a multifunctional protein. Therefore, it is crucial to understand how P1 disrupts the plant’s immune system to promote viral infection.

In this study, WYMV-P1 was identified as a pathogenic protein. We hypothesized that P1 exerts its pathogenic function by disrupting the host’s immune response. Therefore, transcriptomic and proteomic intergative analyses were performed to identify the DEGs and DEPs in WYMV-infected Fielder and overexpressing (OE)-P1 wheat plants. The silencing of peroxidase (*TaPOD*) and thioredoxin reductase (*TaTrxR*) genes suggested that they can positively regulate WYMV infection by influencing ROS accumulation in wheat plants. Exogenous application of hydrogen peroxide (H_2_O_2_) induced resistance to WYMV in wheat plants. This suggests that P1 may promote virus infection by enhancing the expression of *TaPOD* and *TaTrxR* to scavenge ROS. Our study provides a new understanding of the molecular basis of WYMV pathogenicity.

## 2. Results

### 2.1. P1 Is a Virulence Factor That Enhances the Pathogenicity of Viruses

**Figure 1:**
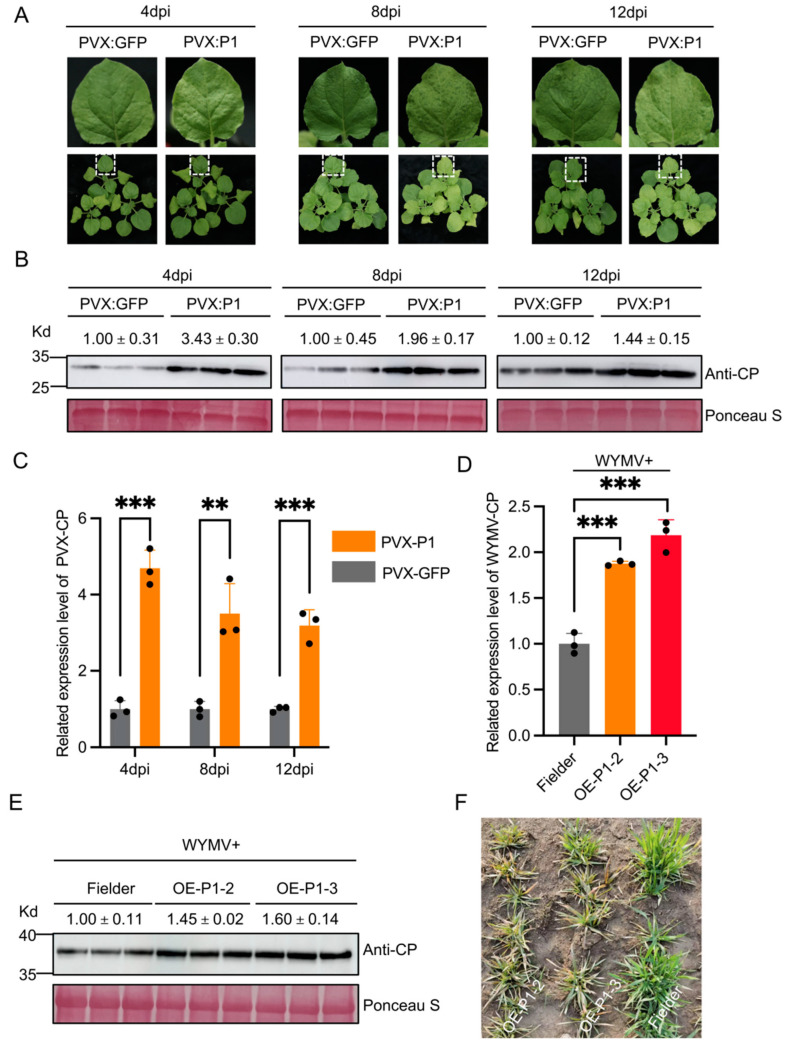
WYMV-P1 is a pathogenic protein that can increase the pathogenicity of PVX in *N. benthamiana* and WYMV in wheat. (**A**) *N. benthamiana* plants were inoculated with PVX:GFP or PVX:P1 through agroinfiltration. Phenotypic symptoms were photographed at 4, 8, and 12 dpi. (**B**) Western Blot analysis was performed using a PVX-CP-specific antibody to detect the accumulation levels of PVX in the *N. benthamiana* leaves infected with PVX-GFP or PVX-P1 at 4, 8, and 12 dpi. Ponceau staining indicates the sample loadings. (**C**) qRT-PCR analysis of mRNA accumulation levels of PVX in the assayed *N. benthamiana* plants using PVX-CP-gene-specific primers. Values are means ± standard deviations (SDs); student’s *t*-test, *n* = 3, *t* = 12.19, 5.36, 8.957; *p <* 0.0003, 0.0058, 0.0009. (**D**) The accumulation levels of the WYMV in OE-P1 #2, OE-P1 #3, and Fielder wheat plants were determined via a qRT-PCR. Values are means ± SDs; student’s *t*-test, *n* = 3, *t* = 12.92, 10.05; *p* < 0.0002, 0.0006. (**E**) Detection of WYMV accumulation levels in plants through Western Blotting using a WYMV-CP-specific antibody. (**F**) Phenotypic symptoms of OE-P1 wheat plants in the nursery containing the WYMV in Yangzhou, Jiangsu, 2024. Fielder wheat plants were used as the control. ** *p* < 0.01, *** *p* < 0.001.

*N. benthamiana* plants were inoculated with PVX:P1 and PVX:GFP. At 4 days post-inoculation (dpi), the phenotypic symptoms in PVX:P1-inoculated *N. benthamiana* plants were slightly stronger than those of *N. benthamiana* plants inoculated with PVX:GFP. Nonetheless, at 8 dpi and 12 dpi, severe mosaic and vein chlorosis was observed in the PVX:P1-inoculated plants. Conversely, the PVX:GFP-inoculated plants exhibited only minor mosaic symptoms and vein chlorosis (Figure 1A). Furthermore, qRT-PCR analysis demonstrated a higher expression level of PVX coat protein (CP) in the systemic leaves of PVX:P1-inoculated plants compared to that of PVX:GFP-inoculated plants, at 4 dpi, 8 dpi, and 12 dpi (Figure 1B), and similar results were obtained via Western Blotting (Figure 1C, Table S1).

Four OE-P1 transgenic wheat lines were identified by PCR with specific primers and Lines 2 and 3 were identified as positive plants (Figure S1). Next, the OE-P1#2 and OE-P1#3 lines were inoculated with WYMV. After 28 dpi, qRT-PCR and Western Blot analysis were performed to detect the accumulation level of WYMV in systemically infected leaves. The findings indicated that the accumulation of WYMV-CP in the OE-P1#2 and OE-P1#3 lines was significantly increased compared to Fielder plants (Figure 1D,E, Table S2). We also observed that the OE-P1 wheat plants showed more severe mosaic symptoms than ‘Fielder’ plants in the nursery containing WYMV (Figure 1F). These results clearly indicate that P1 is a pathogenic factor that promotes WYMV infection.

### 2.2. Transcriptome Analysis of the OE-P1 Transgenic Wheat Plants Under WYMV Infection

**Figure 2:**
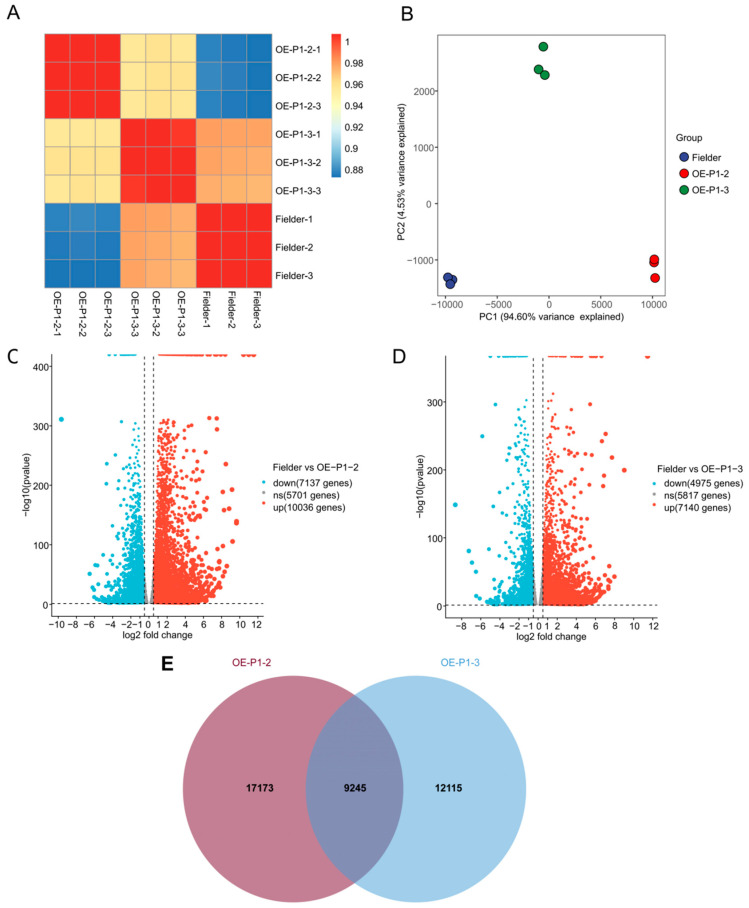
Transcriptomic analysis of OE-P1 transgenic wheat under WYMV infection. (**A**) A heatmap illustrating the Pearson correlation coefficients among samples, with blue indicating a weak correlation and red indicating a strong correlation. (**B**) Principal component analysis was conducted to assess the RNA-seq data’s reproducibility and stability. (**C**) Volcano plot of the downregulated (7137, blue) and upregulated (10,036, red) DEGs in OE-P1#2. (**D**) Volcano plot of the downregulated (4975, blue) and upregulated (7140, red) DEGs in OE-P1#3. (**E**) Statistical Venn diagram of the correlation number of differentially expressed genes (OE-P1#2 vs. OE-P1#3, 9245).

We performed transcriptomic analysis for WYMV-infected Fielder, OE-P1#2, and OE-P1#3 wheat plants. The accumulation of the WYMV was observed via RT-PCR and Western Blotting at 28 dpi (Figure S2A,B). In addition, the upper leaves presented typical mosaic symptoms at 28 dpi (Figure S2C). The symptomatic leaves from Fielder, OE-P1#2, and OE-P1#3 wheat plants were collected to extract RNA for transcriptome analysis. The goal of this analysis was to explore gene expression differences between WYMV-infected OE-P1 wheat plants and WYMV-infected control plants. Pearson’s correlation coefficient showed a positive correlation between groups ranging from 0.88 to 1 (Figure 2A). Moreover, principal component analysis indicated that the degree of clustering between duplicate samples was high (Figure 2B). These findings show that the transcriptome data are reliable. When *p* < 0.05, genes with an average fold change > 0.5 or <−0.5 were considered DEGs (Table S3). In total, 17,173 and 12,115 DEGs were identified in OE-P1#2 and OE-P1#3, respectively (Figure 2C,D), with 9245 DEGs shared between the two (Figure 2E). These results indicate that P1 significantly affects gene transcription in wheat under WYMV infection.

### 2.3. Proteome Analysis of the OE-P1 Transgenic Wheat Plants Under WYMV Infection

**Figure 3:**
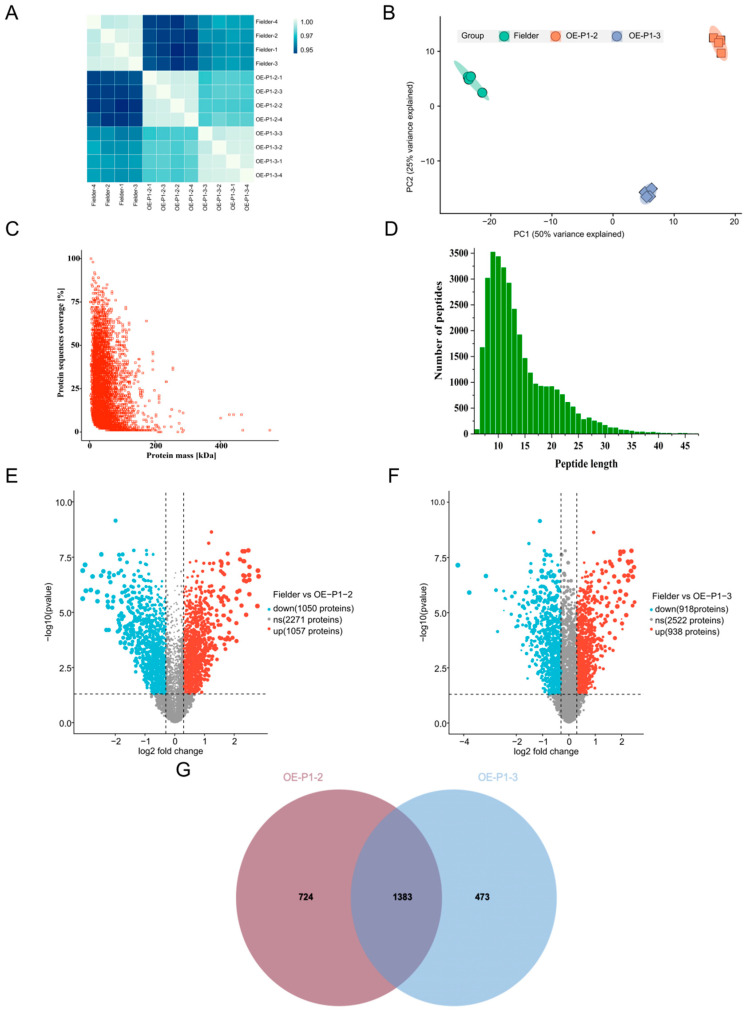
Proteomic analysis of OE-P1 transgenic wheat under WYMV infection. (**A**) A heatmap illustrating the Pearson correlation coefficients among samples. (**B**). Principal component analysis was conducted to assess the LC-MS/MS data’s reproducibility and stability across samples. (**C**) Mass delta values of all identified peptides. (**D**) The distribution of lengths for all detected peptides. (**E**) Volcano plot of the downregulated (1050, blue) and upregulated (1057, red) DEPs in OE-P1#2. (**F**) Volcano plot of the downregulated (918, blue) and upregulated (938, red) DEGs in OE-P1#3. (**G**) Statistical Venn diagram of the correlation number of differentially expressed proteins (OE-P1#2 vs. OE-P1#3, 1383).

We performed a comprehensive proteomic analysis for WYMV-infected Fielder, OE-P1#2, and OE-P1#3 wheat plants. The analysis of the Pearson correlation coefficient indicated a sufficient biological repeat correlation (Figure 3A). In addition, principal component analysis revealed a high degree of clustering between duplicate samples (Figure 3B). These findings indicate that the proteomic data are reliable. A total of 33,622 peptides were identified with an average mass error of <0.02 Da, indicating the high accuracy of LC-MS/MS data (Figure 3C). The identified peptide lengths were mostly between six and twenty amino acid residues, in accordance with the rules of trypsin hydrolysis and HCD fragmentation (Figure 3D), thus meeting the quality control standards. A total of 4378 proteins were identified, of which 2875 were quantified. Proteins with an average fold change of >0.3 or <−0.3 at *p* < 0.05 were considered DEPs (Table S4). A total of 2107 DEPs and 1856 DEPs were identified in OE-P1#2 and OE-P1#3, respectively (Figure 3E,F), with 1383 DEPs shared between the two (Figure 3G). These results indicate that P1 has a significant effect on the whole wheat proteome during WYMV infection.

### 2.4. Integrated Transcriptomic and Proteomic Analysis

**Figure 4:**
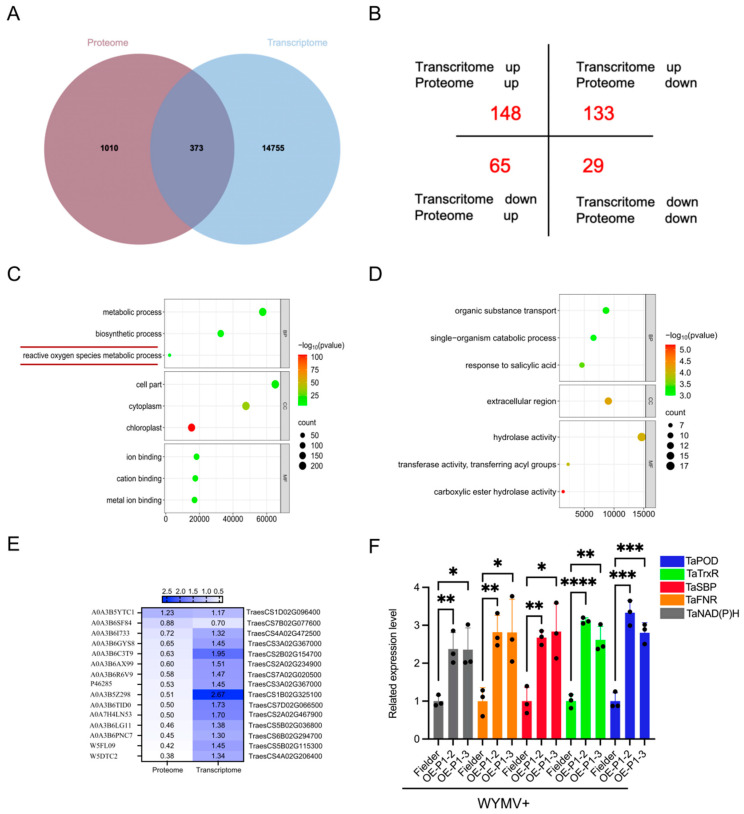
Classification, enrichment, and validation of transcriptome-proteome intersections. (**A**) Venn diagram of transcriptome- and proteome-related quantitative statistics (375). (**B**) Four-quadrant plot of the transcriptome and proteome intersections. (**C**) GO enrichment analysis of upregulation in both the transcriptome and proteome. DEGs/Ps were categorized into the biological process (BP), cellular component (CC), and molecular function (MF) GO classes based on GO line items. (**D**) GO enrichment analysis of downregulation in both the transcriptome and proteome. (**E**) Heatmap of the protein expression of ROS metabolic pathways (15 proteins). (**F**) qRT-PCR was used to verify the expression levels of the five proteins with the highest expression in the ROS metabolic pathway at the transcriptional level. Values are means ± SDs; student’s *t*-test, *n* = 3, *t* = 5.207, 3.991, 5.553, 3.324, 7.136, 3.878, 19.100, 7.023, 10.260, 9.102; *p* < 0.0065, 0.0163, 0.0051, 0.0293, 0.002, 0.0179, 0.0001, 0.0022, 0.0005, 0.0008. * *p* < 0.05, ** *p* < 0.01, *** *p* < 0.001, **** *p* < 0.0001.

InteractiVenn integrated 9245 DEGs from the transcriptome and 1383 DEPs from the proteome and a total of 373 differentially expressed genes and proteins (DEGs/Ps) were identified (Figure 4A, Tables S5 and S6). A total of 373 DEGs/Ps were classified into four types (Figure 4B) and were converted to the ENSEMBL-GENE-ID format (Table S7). There are a total of 148 upregulated DEGs/Ps and 29 downregulated DEGs/Ps in both the proteome and transcriptome, respectively. Additionally, 198 DEGs/Ps tended to be inconsistent in transcriptome and proteome (Tables S8–S11) and Gene Ontology (GO) enrichment analysis was subsequently performed. These DEGs/Ps were categorized into biological processes, cellular components, and molecular functions (Figure 4C,D and Figure S3A,B, Table S12). In terms of biological processes, we found that the genes of the ROS metabolic pathway were significantly upregulated in both the transcriptome and proteome. We generated heat maps of the expression of 15 ROS metabolic pathway proteins from Figure C (Figure 4E, Table S13). Considering that proteins are key to cellular function, we selected five proteins with relatively high expression levels from this pathway and verified their expression levels at the transcriptional level via a qRT-PCR; the results revealed that their expression trends were consistent with the transcriptomic and proteomic data (Figure 4F, Table S14). This suggests that P1 may regulate ROS metabolic pathway genes at the protein level.

### 2.5. TaPOD and TaTrxR Positively Regulate WYMV Infection by Reducing ROS Accumulation

**Figure 5:**
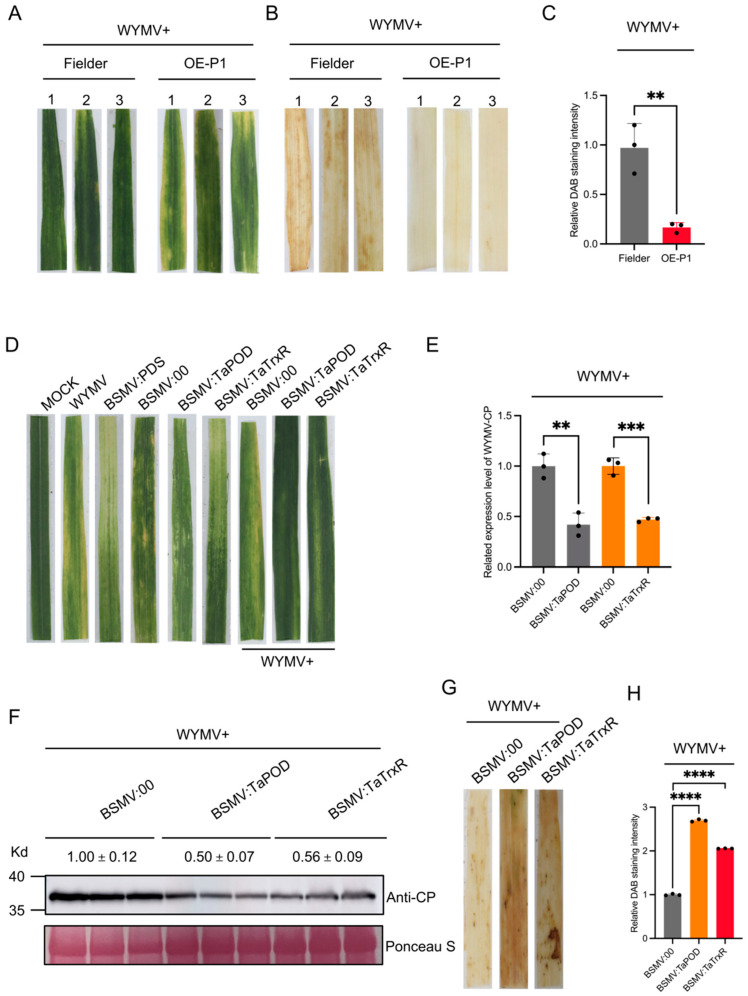
P1 affects ROS accumulation and promotes viral infection and *TaPOD* or *TaTrxR* silencing can increase ROS accumulation and inhibit WYMV infection in wheat. (**A**) Systemic foliar symptoms in WYMV-infected Fielder and OE-P1 wheat plants. Photographs were taken at 28 dpi. (**B**) In situ determination of H_2_O_2_ in WYMV-infected systemic leaves of Fielder and WYMV wheat plants and OE-P1 and WYMV wheat plants. DAB staining was used to analyze H_2_O_2_ production. (**C**) Histograms showing the relative DAB intensity. Each comprised three technical replicates; *t* = 5.538, *p* < 0.0052 (Student’s *t*-test). (**D**) Phenotypes of the fourth leaves of the plants inoculated with phosphate-buffered saline (Mock), WYMV, BSMV:*TaPDS*, BSMV:00, BSMV:*TaPOD*, BSMV:*TaTrxR*, BSMV:00 and WYMV, BSMV:*TaPOD* and WYMV, and BSMV:*TaTrxR* and WYMV. (**E**) qRT-PCR results of WYMV-CP mRNA expression in BSMV:*TaPOD* and WYMV plants and BSMV:*TaTrxR* and WYMV plants. The means ± SDs were calculated from three biological replicates relative to WYMV-infected BSMV:00 plants and each biological replicate comprised three technical replicates; *t* = 14.510, 9.490; *p* < 0.0001, 0.0007 (Student’s *t*-test). (**F**) Western Blot analysis showing the protein expression of WYMV-CP in BSMV:*TaPOD* and WYMV plants and BSMV:*TaTrxR* and WYMV plants. BSMV:00 and WYMV was used as a control. (**G**) In situ determination of H_2_O_2_ in WYMV-infected systemic leaves of BSMV:00 and WYMV, BSMV:*TaPOD* and WYMV, and BSMV:*TaTrxR* and WYMV at 14 dpi. DAB staining was used to analyze H_2_O_2_ production. (**H**) Histograms showing the relative DAB intensity. Each biological replicate comprised three technical replicates; *t* = 29.53, 16.83; *p* < 0.0001, 0.0001 (Student’s *t*-test). ** *p* < 0.01, *** *p* < 0.001, **** *p* < 0.0001.

Because the ROS metabolism pathway was specifically enriched among the DEGs/Ps that were upregulated in both the transcriptome and proteome, we speculated that P1 promotes WYMV infection by affecting host ROS accumulation. Therefore, we inoculated OE-P1 and Fielder plants with WYMV at the two-leaf stage and visible yellow mosaic symptoms appeared on the leaves of the inoculated wheat plants at 28 dpi (Figure 5A). Results revealed that the H_2_O_2_ content in OE-P1 wheat plants was significantly lower than that in Fielder plants (Figure 5B,C). To clarify which host genes are affected by P1 to regulate ROS accumulation, peroxidase (*TaPOD*) and thioredoxin reductase (*TaTrxR*), with the most significant difference in the validation of qRT-PCR, were screened in the enriched ROS metabolic pathway (Figure 4F). These two genes were subsequently silenced by barley stripe mosaic virus virus-induced gene silencing (BSMV-VIGS). The qRT-PCR results revealed that the expression levels of *TaPOD* and *TaTrxR* in the BSMV:*TaPOD*-inoculated and *TaTrxR*-inoculated wheat plants were lower than that in the BSMV:00-inoculated plants at 10 dpi (Figure S4, Table S15). The BSMV:*TaPOD-*, BSMV:*TaTrxR-* and BSMV:00-inoculated wheat plants were then inoculated with WYMV. After 14 dpi, milder mosaic symptoms on the BSMV:*TaPOD*-inoculated and *TaTrxR*-inoculated wheat plants were observed compared to the BSMV:00- and WYMV-inoculated wheat plants (Figure 5D). The qRT-PCR and Western Blot results revealed that the accumulation level of WYMV in the BSMV:*TaPOD*- and WYMV-inoculated and the BSMV:*TaTrxR*- and WYMV-inoculated wheat plants was much lower than that in the BSMV:00- and WYMV-inoculated wheat plants (Figure 5E,F, Table S16). In addition, we determined the ROS concentrations in BSMV:00-inoculated, BSMV:*TaPOD*-inoculated, and BSMV:*TaTrxR*-inoculated wheat plants via DAB staining. The results revealed that the ROS contents were significantly greater in BSMV:*TaPOD*- and BSMV:*TaTrxR*-inoculated wheat plants than in BSMV:00-inoculated wheat plants (Figure 5G,H).

### 2.6. Exogenous H_2_O_2_ Significantly Induced Resistance to WYMV Infection in Wheat

**Figure 6:**
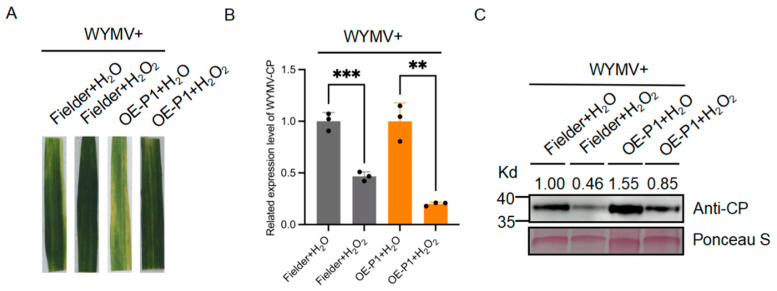
(**A**) Phenotypes of wheat treated with 1 mM H_2_O_2_ and H_2_O after inoculation with WYMV at 28 dpi. (**B**) qRT-PCR results of WYMV-CP mRNA expression in H_2_O-treated Fielder and OE-P1 plants and in H_2_O_2_-treated Fielder and OE-P1 plants. Values are means ± SDs, Student’s *t*-test; *n* = 3; *t* =9.699, 7.809; *p* < 0.0006, 0.0015. (**C**) Western Blot analysis showing the protein expression of WYMV-CP in H_2_O-treated Fielder and OE-P1, H_2_O_2_-treated Fielder, and OE-P1 wheat plants. ** *p* < 0.01, *** *p* < 0.001.

H_2_O_2_ is a marker of ROS and 1 mM H_2_O_2_, thus, was applied to the leaves of Fielder and OE-P1 wheat plants; H_2_O was used as the control. WYMVs were inoculated 12 h after treatment. The results showed that wheat plants treated with H_2_O_2_ displayed slighter mosaic symptoms compared to H_2_O-treated plants (Figure 6A). Additionally, qRT-PCR and Western Blot analysis showed that the expression level of the WYMV in the H_2_O_2_-treated wheat plants was significantly lower compared to that in the H_2_O-treated plants (Figure 6B,C, Table S17).

## 3. Discussion

ROS play a key role in plant immunity [27,28]. Low levels of ROS act as signaling molecules that activate plant defense responses whereas high levels of ROS have direct antibacterial and antiviral activities [1]. However, high levels of ROS can cause damage to the plant itself. To cope with this situation, under normal circumstances, plants maintain ROS homeostasis through a series of antioxidant enzymes, such as peroxidase (POD) and thioredoxin reductase (TrxR) [29]. These two proteins have been reported to scavenge H_2_O_2_ in plants [15]. At present, the high diversity of interactions between ROS and plant viruses is not yet understood. Recently, it has been reported that plant viral proteins can affect plant immunity by influencing antioxidant enzymes [30,31]. This finding is essentially consistent with the results of the present study, which revealed that WYMV-P1 affects the antioxidant proteins TaPOD and TaTrxR, thereby affecting plant immunity.

RNA-Seq has become an indispensable tool for the transcriptome-wide analysis of differential mRNA expression [16]. MS-based proteomics has become a powerful technology for quantifying the entire complement of proteins in cells [32]. Transcriptome and proteome are omics techniques that study cell activity and function at different levels and they are both different and complementary. The integrated analysis of transcriptome and proteome data can provide a comprehensive understanding of the molecular regulatory network in the cell from the two levels of gene expression transcription and translation and reveal the mechanism of biological processes more deeply. In this study, we found that certain genes do not have the same tendency at the transcriptional level as the protein level, which may indicate the existence of a post-transcriptional regulatory mechanism. In contrast, the transcriptome and proteome showed consistent trends and we believe that the results are more reliable. This multi-level analysis helps us dig deeper into the complex network of P1 influencing plant physiological processes. Transcriptome and proteome data can validate and complement each other. We use the transcriptome and proteome of genes that are significantly upregulated for the next step. This joint analysis method provides us with a more accurate and comprehensive research perspective, avoiding the one-sided conclusions that may be produced by a single analysis method.

WYMV-encoded P1 was found to be a pathogenic protein for viral infection (Figure 1A–F) but its specific mechanism of action is still unclear. WYMV-P1 has been reported to promote viral infection in wheat by acting as a VSR via interference with calmodulin-related antiviral RNAi defense mechanisms [22]. We also observed enriched calcium transport-related pathways in the transcriptome GO enrichment data (Figure S5) but not in the transcriptome-proteomic association analysis of the GO enrichment data, possibly due to the VSR occurring only at the transcriptional level and not at the protein level. The regulation of gene expression is a complex, multifaceted process and changes at the transcriptional level are not always directly reflected at the protein level. Post-transcriptional regulatory mechanisms, such as mRNA variable splicing, RNA editing, mRNA stability regulation, translation initiation, and elongation regulation, may result in the lack of a direct correspondence between transcript abundance and protein abundance [33]. However, another factor worth exploring in depth is the inaccuracy of InteractiVenn’s analytical comparisons. As a tool to visualize and analyze the intersection and union relationship between multiple data sets, InteractiVenn has been widely used in many types of research, but it also has certain limitations [34]. First, the quality and integrity of the data have a significant impact on the results of the analysis. Second, the InteractiVenn analysis focused primarily on overlaps between datasets and ignored other features of the data, such as multiples and trends of changes in gene expression or protein abundance. To investigate how the P1 protein affects host gene-to-protein levels during WYMV infection, thereby promoting infection, we sequenced the transcriptomes and proteomes of OE-P1 and Fielder wheat (control) plants. By combining the transcriptome and proteome analyses, the molecular mechanism of P1 in the process of viral infection was investigated. We found that WYMV-P1 can affect *TaPOD* and *TaTrxR* transcription, suggesting that P1 may promote viral infection by interfering with ROS metabolism in plants (Figure 5D,G).

Plant RNA viral infection usually causes symptoms, such as leaf yellowing, necrosis, and plant growth retardation, and leaf yellowing often leads to decreased photosynthetic activity of chloroplasts [35]. In the DEG/P enrichment analysis, we also found that the cellular components of the DEGs/Ps were significantly enriched in chloroplasts (Figure 4C–F). Chloroplasts are the source of numerous defense signals in plant cells and are closely involved in triggering immune initiation but are also among the key sites of ROS metabolic pathways [36].

WYMV-P1 may accelerate ROS scavenging by activating TaPOD and TaTrxR transcription through certain pathways, which may be a favorable strategy for viral infection. When a plant is invaded by a virus, it produces ROS to defend itself against the virus. WYMV-P1 may weaken the plant’s own defense response by affecting the accumulation of ROS in the host plant, thereby creating a favorable environment for the virus’s own infection and proliferation. Exogenous application of H_2_O_2_ to WYMV-inoculated Fielder and OE-P1 wheat showed that the exogenous application of ROS conferred resistance to WYMV in wheat. This mechanism reveals an evolutionary arms race between plant viruses and their host plants, with viruses constantly evolving strategies to evade or suppress plant defense responses. Although this study has several limitations, it provides a foundation for future research and the development of plant virus prevention and control strategies.

## 4. Materials and Methods

### 4.1. Plant Materials and Growth Conditions

Wheat plants, including OE-P1 transgenic wheat, Yangmai158 (a wheat variety susceptible to WYMV), Fielder (a wheat variety with moderate resistance to WYMV), and *Nicotiana benthamiana*, were all grown in a chamber maintaining a temperature of 25 °C, a relative humidity of 70%, and a photoperiod of 16 h light/8 h dark. Post-inoculation with WYMV, the plants were subsequently cultivated in the climate-controlled chamber set at 8 °C for further analysis. To clear the role of P1 during viral infection in plants, we engineered a PVX expression vector carrying the WYMV-P1 (PVX:P1) and used it to infect *N. benthamiana*. A control group was established using a PVX expression vector encoding Green Fluorescent Protein (PVX:GFP). The *N. benthamiana* plants inoculated with PVX were cultivated within a greenhouse environment set at a constant temperature of 25 °C.

### 4.2. Viral Inoculation

Two-leaf plants of the transgenic lines OE-P1#2 and OE-P1#3 were inoculated with WYMV and Fielder wheat plants were inoculated as a control. Fielder, OE-P1#2, and OE-P1#3 RNA were extracted from the infected WYMV. The inoculation of wheat seedlings with in-vitro-transcribed WYMV RNAs was performed as reported previously [21]. Briefly, recombinant plasmids were individually digested using the SpeI and subsequently transcribed in vitro with the Ambion Message Machine Kit from Invitrogen (Waltham, MA, USA). An equivalent quantity of the WYMV RNA1 transcript (5 μg) and RNA2 were mixed in vitro. The mixture of RNA transcripts was then diluted in a 1× FES buffer, which comprised 0.06 M potassium dihydrogen phosphate, 0.1 M glycine, 1% tetrasodium pyrophosphate, 1% feldspar, 1% bentonite, and nuclease-free water adjusted to a pH of 8.5. Subsequently, 10 μL of diluted solution was rub inoculated onto the second leaf of each wheat seedling. Each experimental trial was replicated at least three times, with a minimum of six plants per replication. The sequence of the WYMV-P1 was amplified and, subsequently, inserted into the PVX expression vector. PVX:GFP was employed as the control. Both expression vectors, PVX:GFP and PVX:P1, were then transformed into the *Agrobacterium tumefaciens* strain GV3101. The *Agrobacterium* cultures were incubated overnight and subsequently centrifuged in an infiltration buffer containing 100 mM 2-(N-morpholino) ethanesulfonic acid (MES), pH 5.2, 10 mM magnesium chloride (MgCl_2_), and 200 mm acetosyringone. The OD600 was adjusted to 0.6 and the mixture was infiltrated into the leaves of *N. benthamiana* (three plants per treatment), as previously reported [37].

### 4.3. Transcriptome Sequencing

The monitoring of RNA degradation and contamination was accomplished through the 1% agarose gel electrophoresis. Subsequently, the integrity of RNA was strictly assessed using the RNA Nano 6000 Assay Kit, which was deployed within the Bioanalyzer 2100 System, a product of Agilent Technologies, Inc., a corporation based in Santa Clara, CA, USA. Sequencing libraries were meticulously built according to the Illumina^®^ platform using the NEBNext^®^ Ultra™ RNA Library Preparation Kit (Ipswich, MA, USA), following the manufacturer’s instructions, and corresponding index codes were added to the attribute sequences of each sample. Additionally, mRNA was selectively isolated from the total RNA using poly(t) oligo-conjugated magnetic beads. The lysis procedure was facilitated by the inclusion of divalent cations within the NEBNext First Strand Synthesis Reaction Buffer (5X), which was carried out at elevated temperatures. The synthesis of the first strand of complementary DNA (cDNA) was accomplished through the employment of random hexamer primers in conjunction with the M-MuLV reverse transcriptase enzyme. The subsequent generation of the second strand of complementary DNA was realized through the deployment of DNA polymerase I and RNase H enzymes. Subsequent to the adenylating of the 3′ ends of the DNA fragments, NEBNext adaptors with a hairpin loop structure were appended to facilitate the hybridization procedure. To selectively isolate cDNA fragments ranging from 250 to 300 base pairs in length, the library fragments were subjected to purification via the use of the AMPure XP system, a product provided by Beckman Coulter, Beverly, MA, USA. Subsequently, the cDNA was ligated with 3ul of USER enzyme to select size, procured from New England Biolabs, Ipswich, MA, USA; the mixture was incubated at a temperature of 37 °C for a duration of 15 min and with a denaturation step at 95 °C for 5 min. The polymerase chain reaction (PCR) was performed using the Phusion High-Fidelity DNA Polymerase, with the application of universal PCR primers and index (X) primers. The PCR-generated products underwent a supplementary purification procedure, which was executed with the AMPure XP System. Thereafter, the integrity of the resultant library was evaluated by employing the Agilent Bioanalyzer 2100 system (Santa Clara, CA, USA). The indexed samples were subsequently subjected to clustering on the cBot Cluster Generation System, utilizing the TruSeq PE Cluster Kit v3 cBot HS (Illumina, San Diego, CA, USA), strictly following the manufacturer’s established protocols. Library preparations were sequenced on the Illumina HiSeq platform, resulting in paired-end reads with lengths of 125 base pairs and 150 base pairs, respectively.

### 4.4. Transcriptome Data Analysis

Firstly, the raw reads in fastq format were processed through an internal Perl script. Clean reads were extracted from the raw data by eliminating reads containing adapters, poly-N, and low-quality reads. Simultaneously, the Q20, Q30, and GC content were determined for the clean data, which were then used for all subsequent high-quality analyses. Bowtie v2.2.3 indexed the reference genomes and TopHat v2.0.12 aligned the paired-end clean reads to these indices. TopHat was selected for mapping due to its ability to create a splice junction database from gene model annotations, leading to superior mapping outcomes compared to non-splice-mapping tools. HTSeq v0.6.1 was employed to determine read counts per gene. The FPKM values were calculated for each gene, considering gene length and mapped read numbers. FPKM, which stands for fragments per kilobase of transcript per million mapped reads, accounts for sequencing depth and gene length and is widely used for gene expression quantification [38]. DESeq R package (version 1.18.0) was employed for differential expression analysis across two conditions/groups, each with three biological replicates. Genes with a fold change in the transcriptome greater than 0.5 or less than −0.5 and a *p*–value less than 0.05 are considered differentially expressed genes.

### 4.5. Protein Extraction and Digestion

Protein extraction followed a previously described protocol [39]. Briefly, the wheat leaves were ground into a powdery form using liquid nitrogen and suspended in an extraction solution. This solution contained 40 mM Tris-Cl at pH 8.5, 2 M thiourea, 7 M urea, 10 mM dithiothreitol (DTT), 4% SDS, 2 mM EDTA, and 1 mM PMSF. The suspension was vigorously mixed and then chilled on ice for 10 min; this process was repeated three times. After incubation, the samples underwent centrifugation at 15,000 g for 10 min at a temperature of 4 °C and supernatants were mixed with four volumes of cold acetone and precipitated at −20 °C for at least 10 h. The samples were centrifuged and the sediment was washed using a 1:1 mixture of cold ethanol and acetone three times and redissolved in a buffer with 8 M urea and 100 mM TEAB at pH 8.0. The resolution was incubated for 2 h at room temperature. Protein concentration was measured using a BCA assay. Then, the proteins were reduced with 15 mM DTT at 37 °C for 1 h and alkylated with 50 mM iodoacetamide (IAA) in the dark for 30 min at 25 °C. Excess IAA was removed using additional DTT. Protein extracts were diluted eightfold with 100 mM TEAB and digested with sequencing-grade trypsin at a 1:40 trypsin:protein ratio at 37 °C overnight. An additional aliquot of trypsin (trypsin:protein ratio of 1:50) was then added to ensure complete digestion for 4 h at 37 °C. The digested peptides were desalted using a solid-phase extraction C18 cartridge (Waters, MA, USA) and then lyophilized. The peptide concentration was subsequently measured via a Pierce Quantitative Colorimetric Peptide Assay (Thermo Scientific, Waltham, MA, USA).

### 4.6. LC-MS/MS Analysis and Database Search

To obtain the actual expression of the proteins, we conducted a comprehensive proteome analysis using liquid chromatography-tandem mass spectrometry (LC-MS/MS). The peptides were resuspended in solvent A (0.1% formic acid in water) and then analyzed via a Vanquish Neo UHPLC system coupled with an Orbitrap Eclipse mass spectrometer (Thermo Fisher Scientific) equipped with a nanoelectrospray ion source. A 1 μL aliquot of peptide sample was injected onto a trap column (Thermo Scientific Acclaim PepMap 100, 75 μm × 2 cm, C18, 3 μm, 100 Å) and washed at a flow rate of 10 μL/min for 3 min. Subsequently, the peptides were separated on an analytical column (Thermo Scientific Acclaim PepMap 100, 75 μm × 25 cm, C18, 2 μm, 100 Å) via a linear gradient: 2.5% to 23% solvent B (80% acetonitrile in 0.1% formic acid) over 100 min, followed by an increase to 32% over 20 min, an increase to 90% over 19 min, and finally maintenance at 90% for 10 min. The flow rate through the analytical column was kept at 300 nL/min, with the column temperature adjusted to 50 °C. The electrospray voltage was configured to 2.1 kV and the temperature of the heated capillary was set to 320 °C. The mass spectrometer functioned in a data-dependent acquisition mode, with a cycle time of 1.5 s. Comprehensive MS scans were captured across a mass range of m/z 300–2000 with a resolution of 120,000 and an RF lens setting at 40%. The AGC target was 400,000, with an upper limit of 50 ms for injection time. For MS/MS acquisition, an isolation window of 1.6 m/z was implemented. The AGC target for MS/MS was 50,000, with an upper limit of 54 ms for injection time. The normalized collision energy (NCE) for High Energy Collision Dissociation (HCD) was set to 30%. The peptides were analyzed for label-free quantification using Proteome Discoverer software (Thermo Fisher Scientific, version 2.5). Tandem mass spectra were matched to the wheat proteome database with the following settings: enzyme specificity was set to trypsin, requiring a minimum peptide length of six amino acids and permitting up to two missed cleavage sites. The mass errors for identified peptides’ precursor ions were within 10 ppm and the fragment ion mass tolerance was set at 0.02 Da. Cysteine modification by iodoacetamide was designated as a fixed modification while methionine oxidation and protein N-terminal acetylation were considered variable modifications. A false discovery rate (FDR) threshold of less than 1% was applied at both the peptide and protein levels for acceptance. Furthermore, the data analysis was conducted using the “Wu Kong” platform, which is R-language-based (https://www.omicsolution.com/wkomics/main/ (accessed on 20 March 2024)). For proteins significantly regulated in wheat, those quantified in at least two replicates per treatment group were selected for further analysis. Proteins with a proteomic fold change greater than 0.3 or less than −0.3 and a *p*-value less than 0.05 are considered differentially expressed proteins.

### 4.7. GO Enrichment Analysis

DEGs/Ps were converted to the ENSEMBL-GENE-ID format using the website https://david.ncifcrf.gov/conversion.jsp (accessed on 13 May 2024). Gene Ontology (GO) enrichment analyses of DEGs/Ps were performed using the website http://systemsbiology.cau.edu.cn/agriGOv2/classification_analysis.php?category=Plant&family=Poaceae (accessed on 18 May 2024) and GO terms with *p* values less than 0.05 were considered to be significantly enriched with differentially expressed genes.

### 4.8. Virus-Induced Gene Silencing (VIGS)

To investigate the role of TaPOD and TaTrxR in WYMV infection, *TaPOD* and *TaTrxR* were silenced through a BSMV-VIGS assay. The BSMV-VIGS was performed according to what has been previously reported [40]. The website https://vigs.solgenomics.net was used to predict the conserved TaPOD and TaTrxR fragments. The 300 bp conserved region fragments of TaPOD and TaTrxR were subsequently cloned and inserted into the pBSMVγ vector. The individual linearization of the plasmids pBSMVα, pBSMVβ, pBSMVγ, pBSMVγ:TaPOD, pBSMVγ:TaTrxR, and pBSMVγ:TaPDS was carried out using their respective specific restriction enzymes. After plasmid linearization, RNA transcripts were produced using the mMessage mMachine T7 in vitro transcription kit according to the manufacturer’s protocol (Ambion, Austin, TX, USA). The RNA transcripts corresponding to BSMVα, pBSMVβ, and pBSMVγ (BSMV:00); BSMVα, pBSMVβ, and pBSMVγ:TaPDS (BSMV:TaPDS); or BSMVα, pBSMVβ, and pBSMVγ:TaPOD (BSMV:TaPOD), along with pBSMVγ:TaTrxR (BSMV:TaTrxR), were combined, diluted to a ratio of 3/7 with 1 × FES buffer, and then applied to the second fully developed leaf of each wheat seedling, with an application volume of 10 μL of the transcript mixture per leaf. Plants inoculated with BSMV:00 or BSMV:TaPDS were used as controls. Post-inoculation, wheat seedlings were cultivated in darkness at 28 °C with 70% humidity for 24 h, followed by a 16-h light/8-h dark cycle. After a 10-day period, the plants were re-inoculated with WYMV and maintained in a climate chamber at 8 °C for subsequent analysis.

### 4.9. RNA Extraction and RT-qPCR

Total RNA was extracted from wheat leaves and *N. benthamiana* leaves using the HiPure plant RNA mini-kit (Magen, Guangzhou, China). The extracted RNA was used to synthesize first-strand cDNA using random primers at 1 μg of total RNA per 20 μL of reaction using the First-strand cDNA Synthesis Kit (TOYOBO, Osaka, Japan). Quantitative PCR was performed on an Applied Biosystems QuantStudio 6 Flex system (Applied Biosystems, Foster City, CA, USA) using the SYBR Green qRT-PCR kit (Vazyme, Nanjing, China). Gene expression was quantified using the 2-ΔΔCt method, with triplicate biological samples for each treatment and technical triplicates for each assay. Primer sequences are provided in Supplementary Table S18. In this study, statistical methods, such as mean (Mean), standard deviation (SD), *t*-test, and Analysis of Variance (ANOVA) were used to analyze the difference between the data among the groups.

### 4.10. Western Blot Analysis

For total protein extraction, *N. benthamiana* and wheat tissues were ground and lysed in the buffer with 60% SDS, 100 mM Tris-HCl at pH 8.8, and 2% β-mercaptoethanol. The samples were then subjected to SDS-PAGE and transferred onto nitrocellulose membranes for analysis. The blots were incubated in blocking buffer (5% skim milk and 0.05% Tween 20 in 1 × TBS) for 60 min and then incubated with a specific primary antibody followed by a HRP-coupled anti-rabbit secondary antibody (TransGen Biotech, Beijing, China). The signals were visualized using an Amersham imager 680 (GE Healthcare BioSciences, Pittsburgh, PA, USA), as described in previous studies [40].

### 4.11. DAB Staining

To assess H_2_O_2_ accumulation in wheat and N. benthamiana leaves, they were harvested and stained with a 3,30-diaminobenzidine (DAB) solution. Briefly, harvested wheat leaves were immersed in DAB (1 mg/mL) staining solution overnight and then dipped three to five times in absolute ethanol. As previously reported [41], the relative DAB staining intensity was calculated via ImageJ version 1.54 software (https://imagej.net/ij).

### 4.12. Exogenous Spraying of H_2_O_2_

To further elucidate the possible roles of ROS in response to WYMV infection, exogenous H_2_O_2_ was used to treat Fielder and OE-P1 wheat plants. In total, 1 mM H_2_O_2_ was selected for the exogenous spraying experiment, as previously reported [42]. A total of 12 h before WYMV inoculation, H_2_O and 1 mM H_2_O_2_ were sprayed onto Fielder and OE-P1 wheat plants. The inoculated wheat plants were planted in a chamber with a temperature of 8 °C for an approximate duration of four weeks.

## 5. Conclusions

This study focused on the effect of P1, a pathogenic protein encoded by WYMV, on viral infection. Transcriptomic and proteomic integrative analysis revealed that P1 was involved in the host ROS metabolism pathway. Further studies indicated that P1 activated the expression of oxidoreductase TaPOD and TaTrxR to scavenging ROS, thereby facilitating WYMV infection in wheat. Overall, these results revealed that the WYMV-encoded P1 protein promotes viral infection in wheat by regulating the host ROS metabolic pathway and the expression of related REDOX enzymes, providing an important basis for further understanding of the pathogenic mechanism of the WYMV.

## Data Availability

We have shared the data in the Supplementary Materials file.

## Supplementary Materials

The following supporting information can be downloaded at: https://www.mdpi.com/article/10.3390/ijms26041455/s1.

## Abbreviations

ROS	Reactive Oxygen Species
WYMV	Wheat Yellow Mosaic Virus
POD	Peroxidase
TrxR	Thioredoxin Reductase
SA	Salicylic Acid
JA	Jasmonic Acid
SOD	Superoxide Dismutase
CAT	Catalase
DEGs	Differential Expressed Genes
DEPs	Differential Expressed Proteins
DEGs/DEPs	Differential Expressed Genes/Proteins
VSR	Viral Suppressor of RNA silencing
LC-MS/MS	Liquid Chromatography-tandem Mass Spectrometry
SDs	Standard Deviations
H_2_O_2_	Hydrogen peroxide
PCR	Polymerase Chain Reaction
HCD	High Energy Collision Dissociation
FDR	False Discovery Rate
GO	Gene Ontology
PVX	Potato Virus X
BSMV	Barley Stripe Mosaic Virus
