# Supplementary material for: Wheat Yellow Mosaic Virus P1 Inhibits ROS Accumulation to Facilitate Viral Infection
Source: Int J Mol Sci. 2025 Feb 10;26(4):1455. doi: 10.3390/ijms26041455 (PMC11855546; doi:10.3390/ijms26041455)
Supplement: Supplementary file 1 [file ijms-26-01455-s001.zip › Supplemental Figures.pdf]

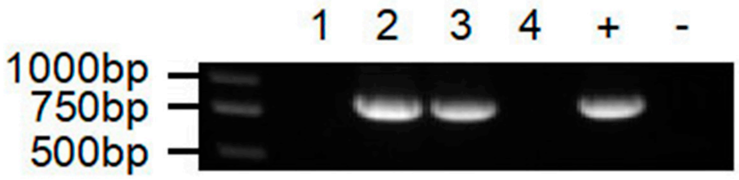

Fig. S1. WYMV P1-specific primers were used to identify overexpressed WYMV P1 transgenic wheat plants lines by PCR.

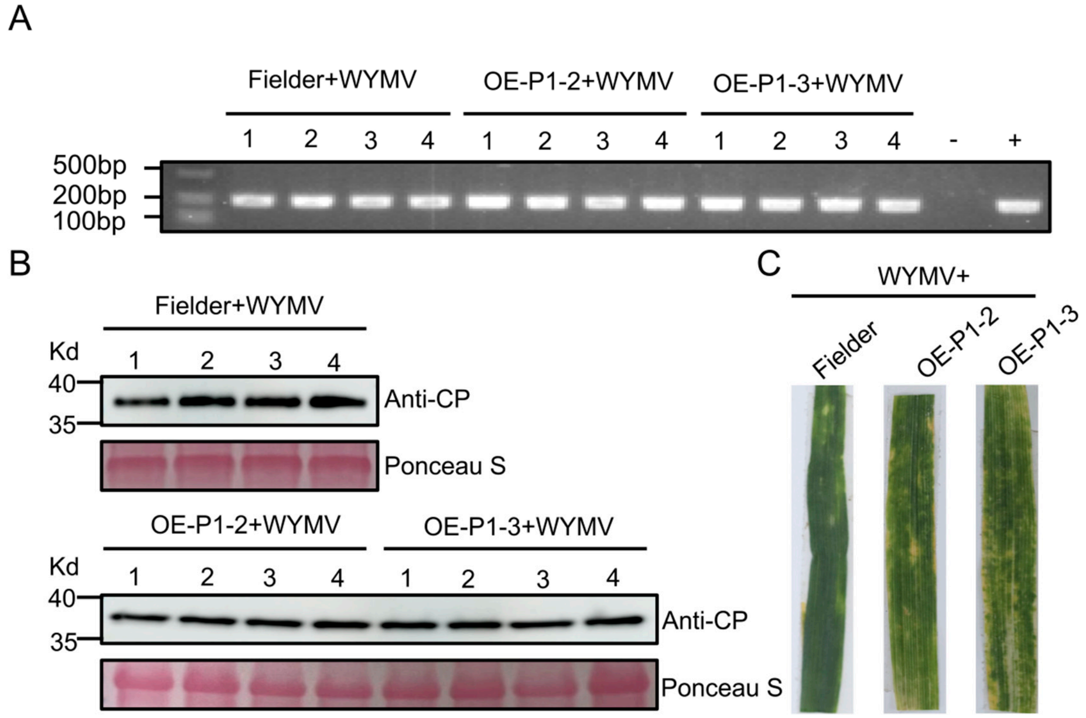

Fig. S2. Detection of WYMV infected wheat. (A) WYMV CP specific primers were used to determine whether wheat was infected with WYMV by PCR. (B) WYMV CP specific antibody were used to determine whether wheat plants was infected with WYMV by Western blot analysis. (C) Leaf symptoms of WYMV-infected Fielder , OE-P1#2 and OE-P1#3 wheat plants. Photos taken at 28 dpi.

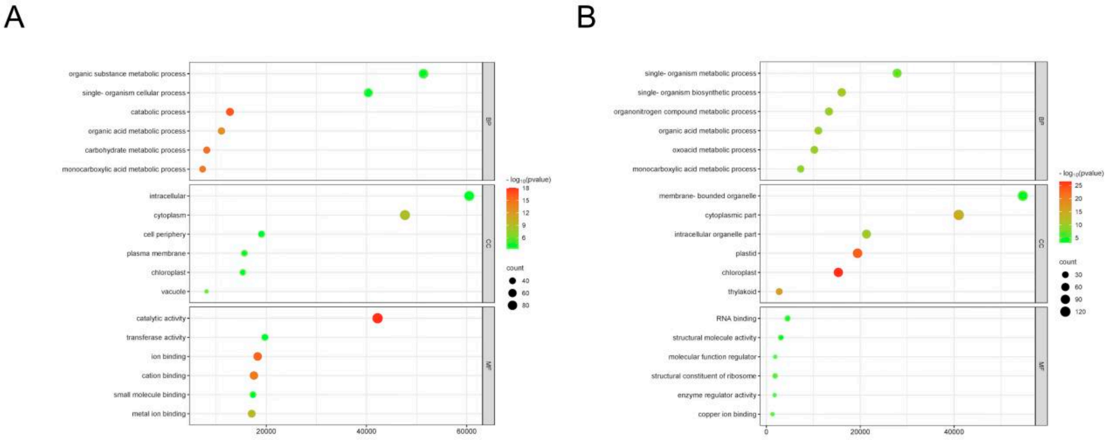

Fig. S3. (A) GO enrichment analysis of transcriptome down-regulated and proteome up-regulation. GOs were categorized into biological process (BP), cellular component (CC), and molecular function (MF) GO classes based on GO line items. (B) GO enrichment analysis of transcriptome up-regulated and proteome down-regulation. GOs were categorized into biological process (BP), cellular component (CC), and molecular function (MF) GO classes based on GO line items.

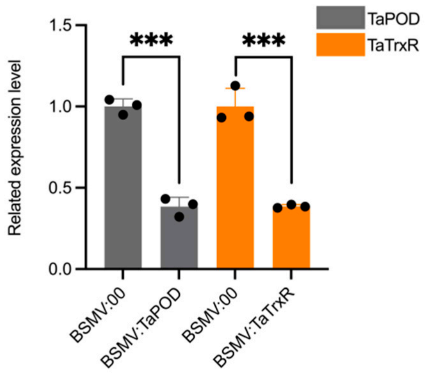

Fig. S4. qRT-PCR results of mRNA expression of *TaPOD* and *TaTrxR* in SMV:TaPOD+WYMV and BSMV:TaTRD+WYMV plants. Means ± SE were calculated from three biological replicates relative to WYMV-infected BSMV:00 plants, and each replicate comprised three technical replicates. ***, $P < 0.01$ (Student's t-test).

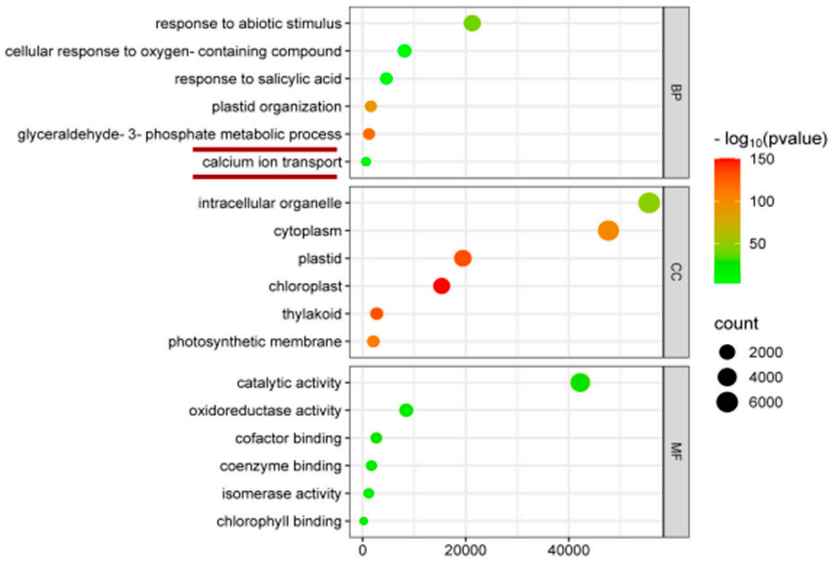

Fig. S5. GO enrichment analysis of transcriptome. GOs were categorized into biological process (BP), cellular component (CC), and molecular function (MF) GO classes based on GO line items.
